# HIF-1α/JMJD1A signaling regulates inflammation and oxidative stress following hyperglycemia and hypoxia-induced vascular cell injury

**DOI:** 10.1186/s11658-021-00283-8

**Published:** 2021-09-03

**Authors:** Min Zhao, Shaoting Wang, Anna Zuo, Jiaxing Zhang, Weiheng Wen, Weiqiang Jiang, Hong Chen, Donghui Liang, Jia Sun, Ming Wang

**Affiliations:** 1 Zhujiang Hospital, Southern Medical University, Guangzhou, 510282 Guangdong China; 2 School of Traditional Chinese Medicine, Southern Medical University, Guangzhou, 510282 Guangdong China

**Keywords:** Diabetes, Vascular disease, Hypoxia-inducible factor-1 alpha, Epigenetics

## Abstract

**Background:**

Endothelial cell (EC) injury accelerates the progression of diabetic macrovascular complications. Hypoxia is an important cause of EC injury. Hypoxia-inducible factor-1 alpha (HIF-1α) is an important hypoxia regulatory protein. Our previous studies showed that high-glucose and hypoxic conditions could upregulate HIF-1α expression and enhance EC inflammatory injury, independently of the nuclear factor kappa-B (NF-κB) pathway. However, it is not clear whether HIF-1α plays a role in vascular disease through epigenetic-related mechanisms.

**Methods:**

We conducted gene expression analysis and molecular mechanistic studies in human umbilical vein endothelial cells (HUVECs) induced by hyperglycemia and hypoxia using RNA sequencing (RNA-seq) and small interfering HIF-1α (si-HIF-1α). We determined HIF-1α and Jumonji domain-containing protein 1 A (JMJD1A) expression by quantitative reverse transcription-polymerase chain reaction (qRT-PCR) and Western blot, analyzed inflammatory protein secretion in the cell supernatant by enzymelinked immunosorbent assay (ELISA), and assessed protein interaction between HIF-1α and JMJD1A by chromatin immunoprecipitation (Ch-IP). We used the Cell Counting Kit8 (CCK-8) assay to analyze cell viability, and assessed oxidative stress indicators by using a detection kit and flow cytometry.

**Results:**

High glucose and hypoxia up-regulated HIF-1α expression, and down-regulated HIF-1α decreased the level of inflammation and oxidative stress in HUVECs. To determine the downstream pathways, we observed histone demethylases genes and related pathway by RNA-sEq. Among these, JMJD1A was the most upregulated gene in histone demethylases. Moreover, we observed that HIF-1α bound to the promoter of JMJD1A, and the ameliorative effects of si-HIF-1α on oxidative stress and inflammatory cytokines in high-glucose and hypoxia-induced HUVECs were reversed by JMJD1A overexpression. Furthermore, knockdown of JMJD1A decreased inflammatory and oxidative stress injury. To determine the JMJD1A-related factors, we conducted gene expression analysis on JMJD1A-knockdown HUVECs. We observed that downregulation of inflammation and the oxidative stress pathway were enriched and FOS and FOSB might be important protective transcription factors.

**Conclusions:**

These findings provide novel evidence that the HIF-1α/JMJD1A signaling pathway is involved in inflammation and oxidative stress in HUVECs induced by high glucose and hypoxia. Also, this pathway might act as a novel regulator of oxidative stress and inflammatory-related events in response to diabetic vascular injury and thus contribute to the pathological progression of diabetes and vascular disease.

**Supplementary Information:**

The online version contains supplementary material available at 10.1186/s11658-021-00283-8.

## Background

The incidence rate of diabetes mellitus (DM) is increasing every year, and shows an increasing trend for younger age of onset [1]. Diabetes as a condition threatening humans has become a worldwide health concern. According to the International Diabetes Federation in 2019, there were nearly 463 million adults with diabetes in the world, and it is estimated that the number of diabetics will exceed seven hundred million by 2045 [2]. Studies have confirmed that long-term diabetes will produce many complications [3], and of these, cardiovascular disease (CVD) is the most common and severe, and its harmful consequences often exceed those of diabetes itself [4, 5]. Studies have confirmed that diabetic patients suffer from 1.45 to 2.99 times the risk of atherosclerosis in non-diabetic patients [6], and the incidence of endothelial cell (EC)-related complications is about 2–4 times higher than in individuals without diabetes [7]. Therefore, it is important for patients with diabetic macrovascular complications to find effective EC protection strategies [8, 9]. However, the molecular mechanisms underlying DM-related macrovascular complication require clarification.

It is generally believed that the response of ECs to a metabolic disorder is the major pathological mechanism underlying the occurrence and development of diabetes-related vascular disease, eventually resulting in endothelial proliferation and arteriosclerotic formation [10]. In addition, inflammation and oxidative stress play an indispensable role in the development of endothelial dysfunction and atherosclerosis in DM [11].

Accumulating evidence in vivo and in vitro has indicated that high glucose levels cause chronic inflammation and oxidative stress, which can increase the expression of various pro-inflammatory cytokines such as interleukin (IL)-6 and IL-8 [12]. Furthermore, in the high-glucose state, activated ECs release pro-inflammatory cytokines produced by local autocrine and paracrine signals between cells, triggering a vicious cycle [13]. In addition, the surface expression of adhesion molecules in ECs, including monocyte chemoattractant protein 1 (MCP-1) and intercellular adhesion molecule-1 (ICAM-1), recruits monocytes that work together with inflammatory cytokines to maintain a continuous state of chronic inflammation and promote endothelial dysfunction [14]. Furthermore, under the stimulation of chronic hyperglycemia, glucose oxidation, nonenzymatic glycation of proteins, and the subsequent oxidative degradation of glycated proteins result in the disproportionate production of free radicals [15]. Antioxidant defense mechanisms can prevent pathological damage and development of insulin resistance caused by hyperglycemia-induced oxidative stress associated with DM [16]. The imbalance between the generation and removal of reactive oxygen species (ROS) by enzymatic and nonenzymatic antioxidant systems induces endothelial dysfunction by increasing endothelium permeability, apoptosis, and necrosis in ECs [17–19]. Accompanying the processes of oxidation and antioxidation, intracellular ROS and malondialdehyde (MDA), as well as antioxidant enzymes superoxide dismutase (SOD), participate in a number of pro-inflammatory pathways in response to high glucose [19]. Together, inflammation and oxidative stress ultimately induce intimal hyperplasia, and play a vital role in the endothelial dysfunction observed in DM. Therefore, it is important to assess cell inflammation and oxidative stress in ECs, as these might facilitate the development of an appropriate therapeutic strategy for diabetic vascular disease.

As the DM progresses, the onset of hypoxic conditions is pivotal. In the high-glucose state, the metabolic supply-demand ratio of affected tissues will undergo profound changes resulting from an increased metabolic demand of infiltrating inflammatory cells coupled with decreased oxygen supply [20]. A major consequence of this is a sharp decline in oxygen utilization, resulting in high-glucose-related hypoxia [21]. Under physio-pathological conditions such as hyperglycemia, inflammation and oxidative stress, hypoxia can further aggravate endothelial injury and trigger a vicious cycle [22–25]. However, although hypoxia is responsible for the macrovascular endothelial dysfunction in DM, the specific mechanisms involved are still unclear.

Hypoxia-inducible factor-1α (HIF-1α), which regulates myocardial hypoxia and initiates the endogenous inflammatory response, is considered to be the key regulator of hypoxic injury [26]. In addition, HIF-1α induces oxidative stress and inflammatory responses to ECs [27], which can result in endothelial dysfunction and severe tissue damage. Studies have shown that HIF-1α can activate NF-κB and enhance the expression of TNF-α under hypoxia [27, 28]. Furthermore, HIF-1α-induced amplification of the NF-κB pathway increases the expression of Toll-like receptors and induces an inflammatory cascade that leads to EC damage [29, 30]. In turn, HIF-1α can be up-regulated during inflammation, while the downregulation of HIF-1α expression can effectively reduce injury caused by inflammation and oxidative stress during hyperglycemia-induced stress in ECs [31, 32]. Therefore, focusing on the HIF-1α axis and its inhibition can help design new therapies for endothelial dysfunction. However, the specific mechanism of HIF-1α-mediated EC protection under hyperglycemia and hypoxia is currently not fully understood.

Jumonji domain-containing protein 1 A (JMJD1A), also called JmjC domain-containing histone demethylation protein 2 A (JHDM2A) or lysine demethylase 3 A (KDM3A), is an important histone H3 lysine 9 (H3K9) dimethyl and monomethyl (me 2/1) demethylating enzyme containing a jumonji domain [33]. JMJD1A is an important epigenetic marker involved in the regulation of transcription factors [34], energy metabolism [35], self-renewal of stem cells [36], spermatogenesis [37], and carcinogenesis [38] by influencing specific gene expression at the level of histone methylation. Studies have shown that demethylated H3K9 increases the transcriptional activity of cancer, and several transcriptional targets related to JMJD1A have been identified, in addition to its important role in the repression of hypoxia-induced transcription [39–43]. In multiple cell lines, JMJD1A was upregulated after hypoxia treatment, which increases hypoxic gene expression [44]. Recent studies have shown that the key hypoxia response element (HRE) in the human JMJD1A gene promoter mediates the up-regulation of JMJD1A through hypoxia [45]. In this study, we mimicked the high-glucose and hypoxia environment, and observed that increased expression of HIF-1α and JMJD1A was induced. However, the specific mechanisms involved in JMJD1A-mediated EC injury under high-glucose and hypoxia are currently unclear. Thus, this study focused on whether HIF-1α and histone demethylase JMJD1A were key participants in mediating the damaging effects of ECs on inflammation and oxidative stress in hyperglycemia and hypoxia and on their interaction.

## Materials and methods

### Cell culture

Human umbilical vein endothelial cells (HUVECs) were purchased from the Chinese Academy of Science Cell Bank (Shanghai, China). The cells were cultured in Dulbecco’s modified Eagle’s medium F12 (DMEM/F12) (Gibco, USA) supplemented with 10% fetal bovine serum (FBS) (Hyclone, USA) and maintained at 37 °C in a humidified atmosphere of 95% air and 5% CO_2_. For the control group, HUVECs were exposed to DMEM/F12 supplemented with 10% FBS and PBS. For hyperglycemic and hypoxic stimulation, cells were exposed to high glucose (25 mM D-glucose) and hypoxia (94% N_2_, 5% CO_2_, and 1% O_2_) in a humidified hypoxia chamber (Coy Laboratory Products) for 48 h.

### RNA interference by siRNA

HUVECs were grown to 50–60% confluency before transfection. Approximately 50 nM of the small interfering RNA (siRNA) targeting HIF-1α or siRNA control (RiboBio, Guangzhou, China) were transfected into HUVECs using the Lipofectamine 2000 (Invitrogen, USA) reagent according to the manufacturer’s instructions. At 24 h after transfection, the cells were treated with high glucose and hypoxia, and the expression of the corresponding genes was tested as described above. RNA interference by si-HIF-1α was confirmed by quantitative RT-PCR (Takara, Japan).

### Lentiviral JMJD1A knockdown and overexpression

HUVECs were subjected to lentiviral transduction using lentiviral particles (shRNA Lentiviral Transduction; Umine Biotechnology Co., Ltd., Guangzhou), with a sequence targeting human JMJD1A (clone ID human NM_018433, sequence, GATCCCCCTAATAACTGTTCAGGAAACTTCCTGTCAGATTTCCTGAACAGTTATTAGGGTTTTTG). The human JMJD1A lentiviral vector (JMJD1A OE group) and the empty lentiviral vector (JMJD1A vector group) were constructed by Hanbio Company (China). Transfectants were placed in puromycin-containing media within 3 days of transduction to select the transduced cells. The cells were passaged until they could be cultured under hyperglycemic and hypoxic conditions.

### Cell viability

Cell viability was assessed using the Cell Counting Kit8 (CCK-8) kit ((#CK04-100 T; Solarbio) according to the manufacturer’s recommendations. A 10 µL volume of CCK-8 solution was added to each well, and the cultures were incubated at 37 °C for 1 h. After measuring the absorbance at 450 nm using a microplate reader, the results were presented as the percentage of viable cells relative to the control group. Each experiment included six readings for each experimental condition.

### RNA isolation and quantitative reverse transcription-polymerase chain reaction

Total RNA was extracted from HUVECs by the TRIzol method and then cDNA was synthesized using a PrimeScript RT Reagent Kit (Takara, Japan) according to the manufacturer’s instructions. Quantitative reverse transcription-polymerase chain reaction (qRT-PCR) was performed using the TaqMan method (Takara, Japan) to measure the mRNA levels of the genes. The qPCR master mix contained 10 µM PCR forward primer and 10 µM antisense primer, ROXII and TB Green reagents. After each measurement, melt curve analysis was performed. All qRT-PCR tests were standardized to β-actin levels. All primers used for qRT-PCR are listed in Additional file 2: Table S1.

### Immunoblotting

HUVECs were lysed on ice with RIPA buffer (Sigma-Aldrich) supplemented with protease and phosphatase inhibitor cocktails (Roche, Germany). After the cell lysate was centrifuged at 16,000*g* for 10 min at 4 °C, solution A and solution B were calculated and prepared, and the total protein concentration was determined by the Bio-Rad protein assay. Then the cell lysate was boiled at 105 °C for 10 min to denature the protein. Next, the protein extracts were electrophoresed on an SDS-PAGE gel and transferred to a polyvinylidene fluoride membrane. Then, the membrane was blocked with 5% bovine serum albumin at 26 °C for 1 h, and incubated with each primary antibody overnight against ICAM-1 (Affinity, AF6088), IL-8 (Affinity, DF6998), MCP-1 (Affinity, DF7577), IL-6 (Affinity, DF6087), HIF-1α (Novus, NB100-105), JMJD1A (Proteintech, 12835-1-AP), and β-actin/GAPDH (Proteintech, 66009-1-lg/60004-1-Ig) as the loading control. After adding 5% BSA horseradish peroxidase-conjugated secondary antibodies to the membrane, it was incubated slowly at 25 °C for 120 min, and exposed via enhanced chemiluminescence. Quantification of the intensity of the protein bands were performed with ImageJ.

### Enzyme-linked immunosorbent assay for detection of IL‑6, IL‑8, ICAM-1, and MCP‑1 in the culture supernatant

After HUVECs were stimulated by hypoxia and high glucose, an equal volume of cell supernatant was collected and centrifuged at 300*g* at 4 °C for 10 min to remove the sediment. The levels of IL-6 (70-EK106/2-96, MultiSciences), IL-8 (70-EK108-96, MultiSciences), ICAM-1 (70-EK189-96, MultiSciences), and MCP-1 (70-EK187-96, MultiSciences) in culture media were measured and analyzed according to the instruction manual of the enzyme-linked immunosorbent assay (ELISA) kit.

### Examination of intracellular ROS generation

HUVECs were washed twice with PBS and collected in an EP tube. Subsequently, the DCFH-DA solution was added to serum-free culture medium to 10 µmol/L. At least 1 ml of the DCFH-DA solution was added to each tube of the cells, and it was incubated at 37 °C for 20 min. Then HUVECs were washed 3 times with serum-free culture medium and centrifuged to completely remove the DCFH-DA solution that did not enter the cells. Then the solutions of each group were transferred to the flow cytometer to measure the mean fluorescence intensity (MFI).

### Measurement of SOD activity and MDA level

To detect the total antioxidant and oxidant capacity, malondialdehyde (MDA) levels and superoxide dismutase (SOD) activity were detected. Following the indicated treatments, the HUVECs were washed using PBS and sonicated for 10 s, at intervals of 20 s, 4 times. The lysate was centrifuged at 14,000 g for 5 min at 4 °C to collect the supernatant. The protein content was measured using a bicinchoninic acid (BCA) kit (Beyotime Institute of Biotechnology, Nantong, Jiangsu, China). The SOD activity and MDA levels were both detected according to the manufacturer’s protocol (SOD: A00-1; MDA: A003-1, Jiancheng Bioengineering Ltd., Nanjing, China).

### RNA isolation, library construction and sequencing RNA

Total RNA was isolated with TRIzol (Takara, Japan). A total of 2 µg of isolated RNA per sample was used for the DNA library preparation. Sequencing libraries were generated using NEBNext Ultra RNA Library Prep Kit for Illumina (NEB, Ipswich, Massachusetts, USA) following the manufacturer’s instructions. Libraries were pooled in equimolar amounts and sequenced (HiSeq 2000, Illumina) using 2 × 132 bp chemistry. RNA-sequencing (RNA-seq) data reported in this study have been deposited in the NCBI’s Gene Expression Omnibus.

### RNA-seq data analysis

Raw read counts were passed through quality control and filtered to obtain high-quality clean reads. The clean reads were mapped to *Homo sapiens* genome Ensembl GRCh38 using HISAT2. Follow-up analysis was carried out after the quality control was qualified. HTseq was used to calculate gene expression, and genes with a fold change > 2 and a false discovery rate p-value < 0.01 were considered as differentially expressed genes (DEGs). The functions of DEGs were summarized using GOseq and KOBAS software. The JMJD1A-related transcription factors were predicted using the AnimalTFDB2 database.

### Chromatin immunoprecipitation (Ch-IP)

Ch-IP assays were performed with HUVECs using standard protocols. Approximately 1 × 10^7^ cells were exposed to air (21% O_2_) or hypoxia (1% O_2_) for 24 h. Cells were crosslinked in a solution of 1% formaldehyde with gentle shaking for 10 min at 26 °C. The cross-linking was stopped by adding 0.125 M glycine and incubating at 37 °C in a hypoxia incubator for 5 min. Then the cells washed twice with pre-cooled PBS, resuspended in RIPA lysis buffer supplemented with protease inhibitors (Pierce), and incubated on ice for 10 min. The crude nuclear lysate was collected by centrifugation at 1000 g, resuspended in lysis buffer II (50 mM Tris HCl, pH 8.1, 1% SDS, 5 mM EDTA) with HALT protease inhibitor mixture, and incubated on ice for 10 min. Cell lysates were sonicated for 10 s, at intervals of 10 s, 15 times. Chromatin was incubated at 4 °C overnight with mouse lgG (control; Santa Cruz) and mouse monoclonal antibody against HIF-1α (Novus Biologicals). The immunoprecipitated DNA was collected by reverse cross-links and purification, and the product DNA was subjected to real-time quantitative PCR reaction (KangChen Bio-tech, Shanghai, China). In this experiment, the primer sequence F: CCTACGCGGTTGAAGAACAA; R: AGACTGAATGGCGAGACCCT was designed for the promoter region of JMJD1A. Then the reaction products were electrophoresed on a 5% polyacrylamide gel, and the Image Quant software was used for quantitative analysis.

### Statistical analysis

Statistical analyses were performed using Prism 7, with unpaired t tests or one-way ANOVA and posttest Tukey’s correction for all analyses. A *p-*value < 0.05 was considered statistically significant.

## Results

### High glucose decreased cell viability and increased inflammation and ROS production in HUVECs

**Fig. 1:**
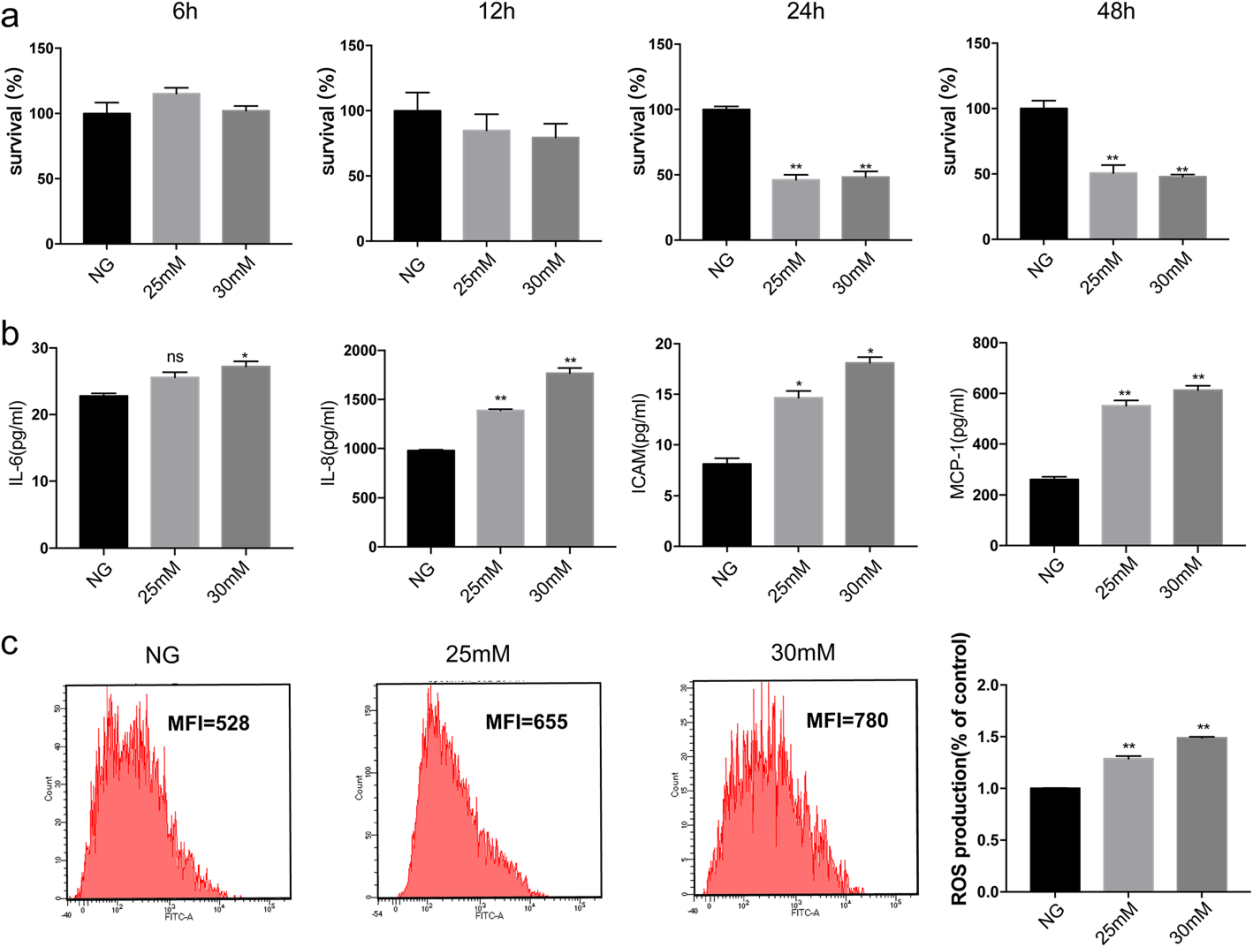
Stimulation with high glucose decreases cell survival and increases ROS levels in HUVECs. **a** Cells treated with various concentrations of glucose (25 or 30 mM) for 6, 12, 24, or 48 h reduced cell viability. High glucose (25 or 30 mM) decreased (**b**) cell viability in a dose- and time-dependent manner, **c** increased IL-6, IL-8, ICAM-1, and MCP-1 protein secretion by ELISA, and **c** increased ROS production compared with control. n = 3; *p < 0.05 and **p < 0.01 vs. control; NG, control

HUVECs exposed to different concentrations of high glucose (25 and 30 mM) exhibited lower cell survival, with 53.77 and 51.69% of cells surviving relative to normal conditions for 24 h and 49.29 and 52.1% for 48 h, respectively, showing the significantly cytotoxic effect of high glucose (*p* < 0.05, n = 3) (Fig. 1a and Additional file 1: Figure S1). Furthermore, to assess the inflammatory status of HUVECs after high-glucose- and hypoxia-induced injury, we first sought to determine the effects of the two different glucose concentrations on protein secretion of IL-6, IL-8, ICAM-1, and MCP-1. As shown in Fig. 1b, treatment with various concentrations of high glucose for 48 h increased IL-6, IL-8, and MCP-1 protein secretion (by 1.12-, 1.41-, and 2.20-fold at 25 mM glucose and 1.06-, 1.80-, and 2.36-fold at 30 mM glucose) compared with controls. Similarly, various concentrations of high glucose also affected ROS production in HUVECs. Compared with untreated normal cultures, 48 h stimulation with high glucose (25 and 30 mM) resulted in a 1.24- and 1.47-fold promotion of ROS production (*p* < 0.05, n = 3) (Fig. 1c). In addition, high-glucose treatment at 25 and 30 mM produced similar increases. Therefore, cells were treated under high-glucose conditions for 25 mM in subsequent experiments.

### Hypoxia enhanced high-glucose-induced inflammation and oxidative production in HUVECs

**Fig. 2:**
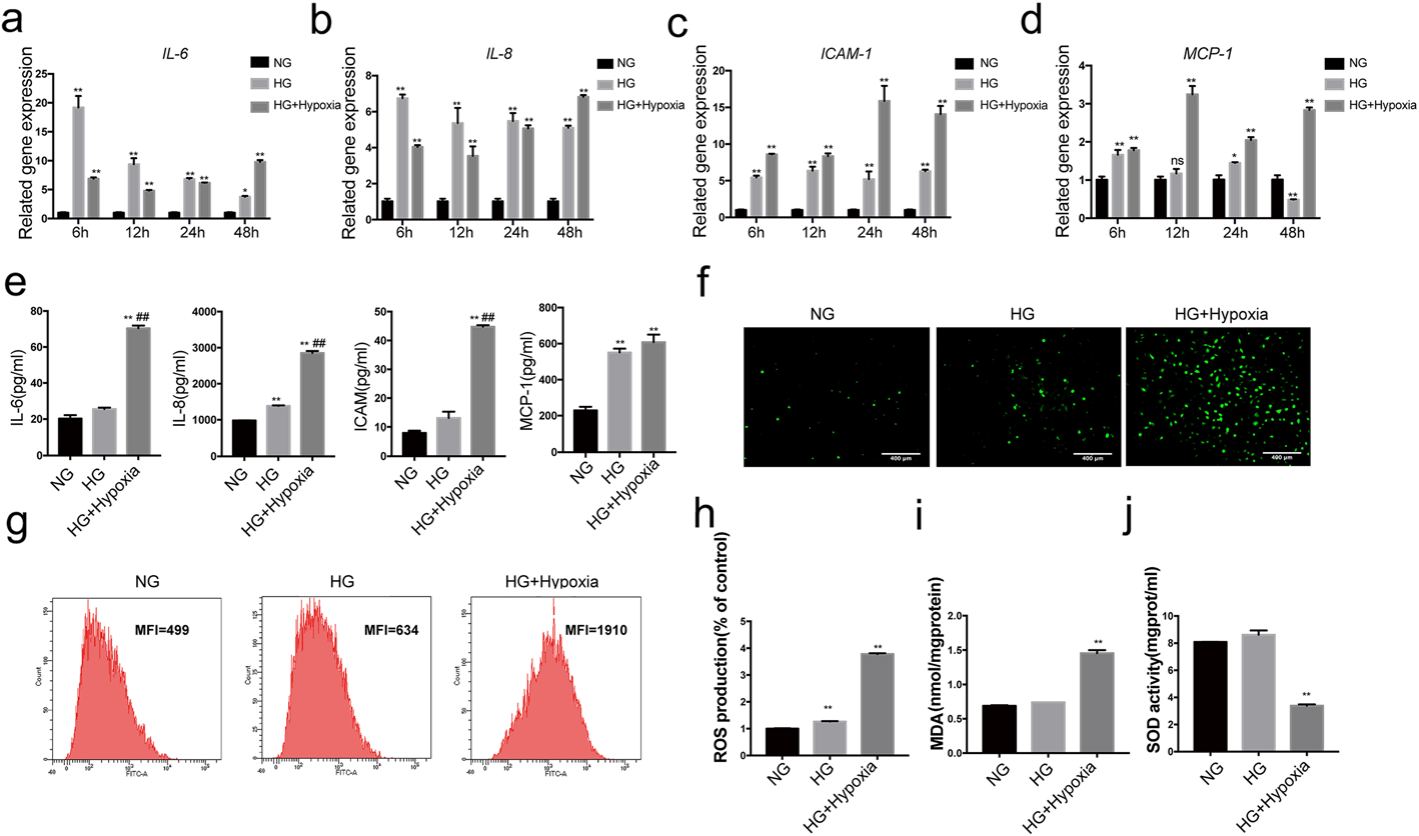
Combined stimulation with high glucose and hypoxia increases inflammation and oxidative stress in HUVECs. Cells were treated with high glucose (25 mM) or with combined exposure to high glucose and hypoxia. Glucose alone or combined stimulation with both factors significantly increased mRNA expression of IL-6 (**a**), IL-8 (**b**), ICAM-1 (**c**), MCP-1 (**d**) after 6, 12, 24, 48 h, and similarly increased protein secretion at 48 h (**e**), increased cell ROS production (**f–h**), increased MDA level (i), and reduced SOD activity (**j**) for 48 h compared with controls. n = 3; *p* < 0.05 and **p < 0.01 vs. control. #*p* < 0.05 vs. glucose. HG, high glucose; HG + Hypoxia, combined stimulus with high-glucose and hypoxia; NG, control

To analyze the pathogenic role of hypoxia in diabetic vascular injuries, a HUVEC high-glucose and hypoxia model was established. We assayed the inflammation of HUVECs induced by high glucose (25 mM) for 6, 12, 24, and 48 h. Our results showed that high glucose affected IL-6 (by 19.2-, 9.39-, 6.83-, 3.75-fold), IL-8 (by 6.75-, 5.37-, 5.49-, 5.10-fold), ICAM-1 (by 5.43-, 6.35-, 5.21-, 6.29-fold), and MCP-1 (by 1.66-, 1.17-, 1.45-, 0.49-fold) mRNA expression in HUVECs cultured for 6, 12, 24 and 48 h compared with controls. Next, we addressed the effects of the combination of the two stimuli on the mRNA expression of inflammatory factors. Compared with the combined stimulus, 6 and 12 h stimulation with high glucose significantly increased the protein secretion of IL-6 (to 2.79- and 1.95-fold) and IL-8 (to 1.67- and 1.52-fold), respectively. However, a large, time-dependent increase in IL-6, IL-8, ICAM-1, and MCP-1 mRNA expression compared with the control or glucose was observed at 48 h. Specifically, the combined stimulus increased mRNA expression of IL-6 (by 2.60- and 3.75- fold), IL-8 (by 1.34- and 6.82-fold), ICAM-1 (by 2.24- and 14.08-fold), and MCP-1 (by 5.84- and 2.84-fold) significantly, compared with the control or high glucose for 48 h, respectively (Fig. 2a–d). Meanwhile, we addressed the effects of the single and combination stimuli for 48 h on the protein secretion of inflammatory factors. A single stimulus with glucose induced IL-8 and MCP-1 protein secretion by 1.40- and 2.40-fold relative to the control, although IL-6 and ICAM-1 protein secretions were not significantly affected. Importantly, the combined stimulus increased the secretion of IL-6, IL-8, ICAM-1, and MCP-1 by 3.48-, 2.90-, 5.52-, and 2.65-fold, respectively, compared with controls, and induced a 2.75-, 2.06-, and 3.42-fold increase in IL-6, IL-8, and ICAM-1 protein secretion compared with glucose alone (Fig. 2e). These findings indicated that a regulatory network involving inflammatory factors might participate in HUVEC damage induced by these two factors. All cells were treated under hypoxic conditions for 48 h in subsequent experiments.

Conversely, our study showed that simultaneous incubation with high glucose (25 mM) and hypoxia for 48 h increased ROS production and MDA levels (by 3.82- and 2.14-fold) compared with controls, and decreased SOD activity by 0.40-fold, respectively (Fig. 2f–j). Overall, these results suggested that high-glucose and hypoxic conditions could increase inflammation and the expression of oxidative stress factors in HUVECs, particularly during simultaneous exposure to both stimuli.

### Inhibition of HIF-1α in hyperglycemia-hypoxia decreased inflammation and oxidative stress in HUVECs

**Fig. 3:**
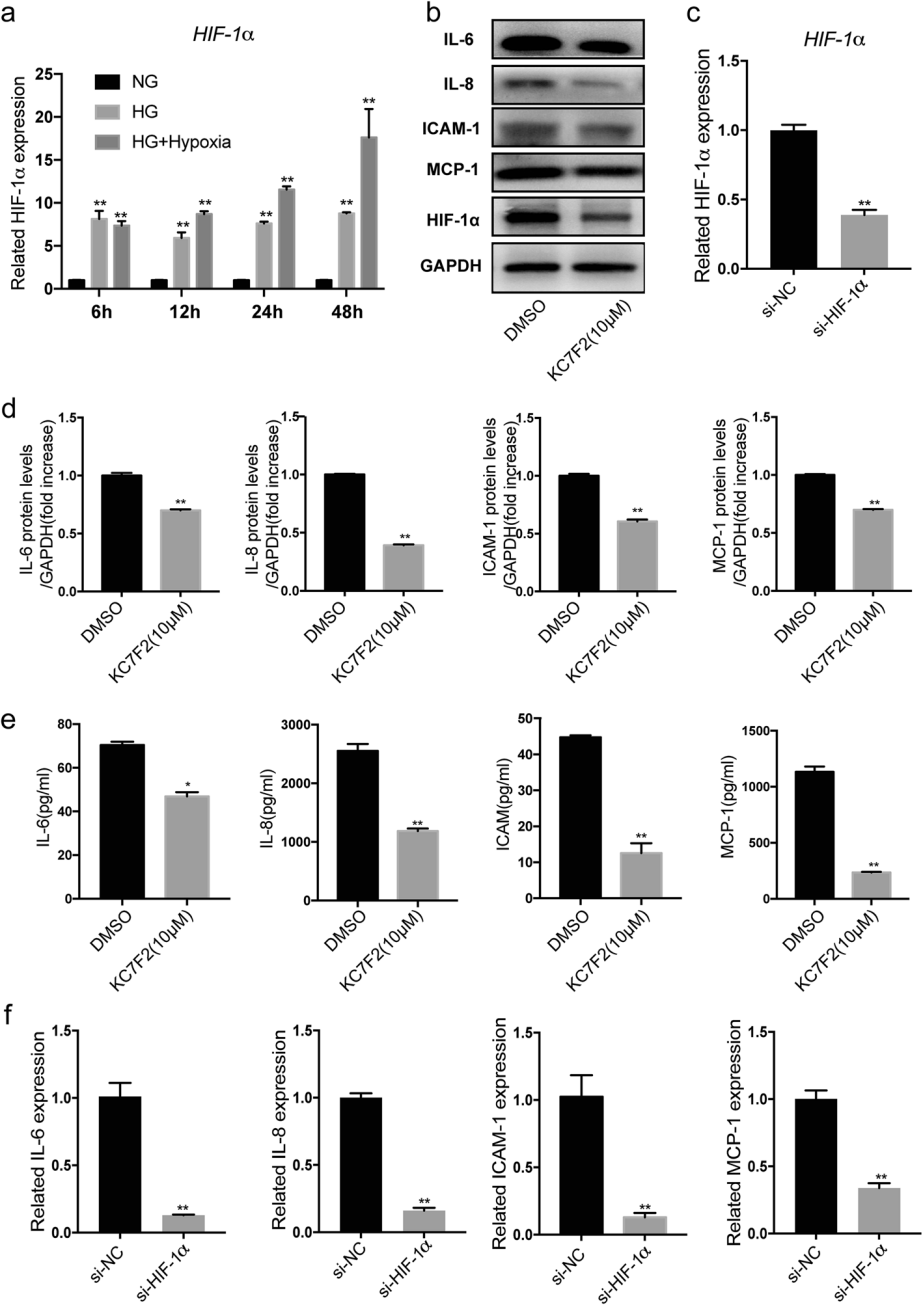
Effects of inhibition of HIF-1α on expression of inflammation after hyperglycemic and hypoxia. **a** Cells were treated with glucose (25 mM) or with combined exposure to high glucose and hypoxia for 6, 12, 24, and 48 h. Exposure to glucose alone or combined stimulation with hypoxia significantly increased gene expression of HIF-1α. **b** Cells were treated with KC7F2 (10 µM) or si-HIF-1α for 48 h following combined stimulation, and IL-6, IL-8, ICAM-1, MCP-1, and **c** HIF-1α levels were analyzed. The relative density of IL-6, IL-8, ICAM-1, and MCP-1 (**d**) was normalized according to GAPDH expression. Importantly, the downregulation of HIF-1α decreased the secretion of IL-6, IL-8, ICAM-1 and MCP-1 based on ELISA and qRT-PCR assays (**e**–**f**). n = 3; *p < 0.05 and **p < 0.01 vs. DMSO. DMSO-treated cells, DMSO; HIF-1α inhibitors KC7F2 (10 µM)-treated cells, KC7F2 (10 µM); si-HIF-1α, small interfering RNA HIF-1α; HG, high glucose; HG + Hypoxia, combined stimulus with high glucose and hypoxia; NG, control

HIF-1α is a hypoxia regulatory protein that plays an essential role in regulating the process of inflammatory and oxidative stress in hypoxia. To assess the role of HIF-1α during hypoxic progression in HUVECs after high-glucose- and hypoxia-induced injury, we first tried to verify the effects of the two stimuli individually or in combination with HIF-1α expression. In our study, HUVECs exposed to 6, 12, 24, and 48 h of glucose (25 mM) exhibited an increase in HIF-1α mRNA levels of 8.15-, 5.93-, 7.63-, and 8.78-fold compared with controls, respectively. The simultaneous incubation with high glucose (25 mM) followed by exposure to hypoxia for 24 and 48 h increased HIF-1α levels (by 1.51-, 11.55-, 2.00-, and 17.62-fold at the mRNA level) compared with high glucose or controls, respectively. However, when cells were treated for 6 and 12 h, the combined stimuli increased HIF-1α expression (7.37- and 8.70-fold at the mRNA level) compared with controls, but there was no statistically significant difference compared with high glucose (Fig. 3a). These results suggested that high glucose and hypoxia could induce HIF-1α expression in cultured HUVECs, particularly during simultaneous exposure to both stimuli.

**Fig. 4:**
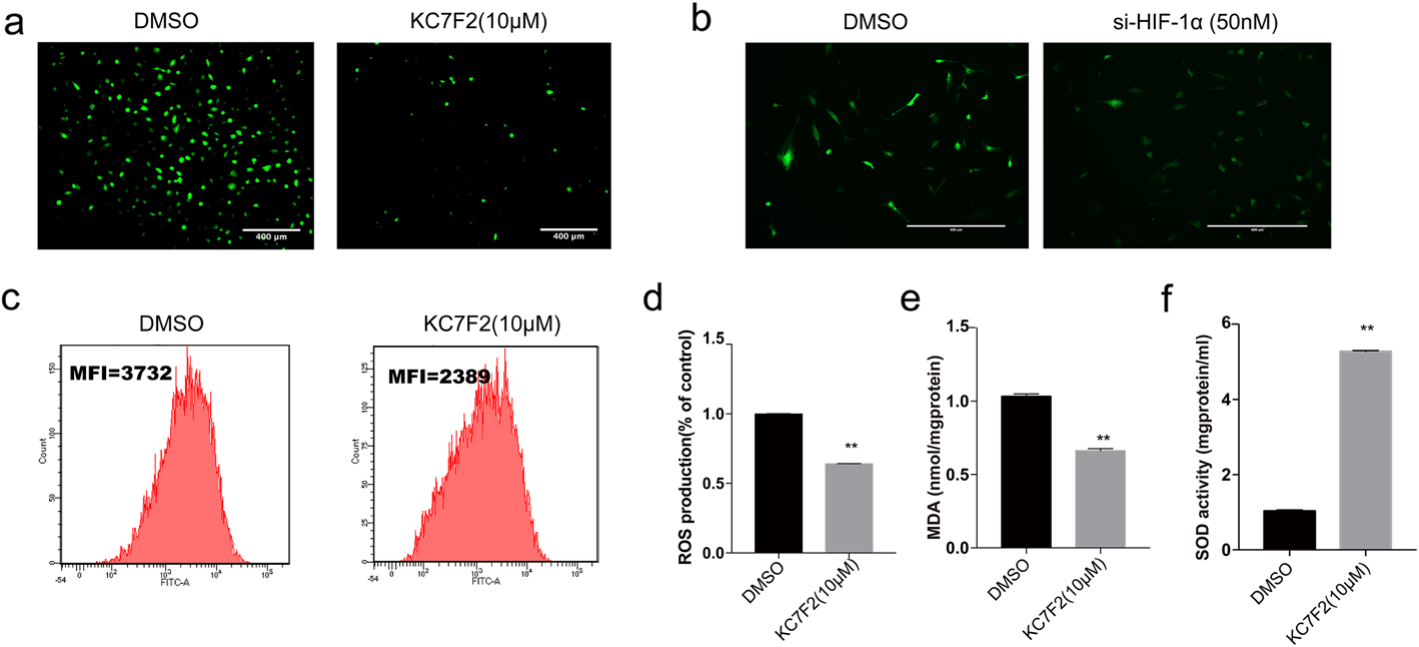
Effects of inhibition of HIF-1α expression on oxidative stress under hyperglycemia and hypoxia. Assays showing ROS (**a–d**), MDA (**e**) and SOD activity (**f**) compared with control. Downregulation of HIF-1α decreased expression of ROS and MDA, and increased SOD activity significantly compared with the control group following combined stimulation. n = 3; *p < 0.05 and **p < 0.01 vs. DMSO. DMSO-treated cells, DMSO; HIF-1α inhibitors KC7F2 (10 µM)-treated cells, KC7F2 (10 µM); si-HIF-1α, small interfering RNA HIF-1α; HG, high glucose; HG + Hypoxia, combined stimulus with high glucose and hypoxia; NG, control

It was previously reported that HIF-1α was closely associated with cell inflammatory and oxidative stress proteins. In order to clarify the potential regulatory network related to HIF-1α after exposure to high glucose and hypoxia, a cell high-glucose and hypoxia model was established. Cells were then treated with a specific inhibitor of HIF-1α KC7F2 and siRNA HIF-1α. The relative protein and gene expression levels of inflammatory factors were analyzed by qRT-PCR, western blotting, and ELISA tests under hypoxic and high-glucose environments (Fig. 3a–f). The results revealed that the protein secretion of IL-6, IL-8, ICAM-1, and MCP-1 in the KC7F2 and si-HIF-1α groups decreased significantly in comparison to that of the DMSO and empty vector-treated groups after the combined stimulus (Fig. 3a–f). In addition, the downregulation of HIF-1α reduced ROS production and MDA release (by 0.64- and 0.66-fold) respectively, and increased SOD activity by 5.28-fold compared with the DMSO-treated group (Fig. 4a–f). These results suggested that downregulation of HIF-1α inhibited inflammation and oxidative stress in HUVECs exposed to combined stimulus-mediated injury. Therefore, these data supported the hypothesis that HIF-1α might accelerate cell inflammation and oxidative stress injury under hyperglycemic and hypoxic conditions.

### RNA sequencing revealed that JMJD1A could be a potential epigenetic regulator in endothelial injury induced by hyperglycemia and hypoxia

To uncover the underlying mechanisms by which HIF-1α promoted cellular oxidative stress and inflammatory progression, we analyzed the gene expression profiles of high glucose and hypoxia by using high-throughput RNA sequencing. Three samples of each group were sequenced. A total of 269,878,950 reads in the control group (on average 89,959,650 reads per sample, ranging from 41,113,776 to 123,552,542 reads) and 125,009,922 reads in the high-glucose and hypoxia group (average 41,669,974 reads per sample, range from 39,797,748 to 43,600,994 reads) passed the quality control. A total of 96.33% of total reads were mapped to the *Homo sapiens* genome Ensembl GRCh38. 24,366 genes with counts greater than 10 in six samples were identified for further analysis.

**Fig. 5:**
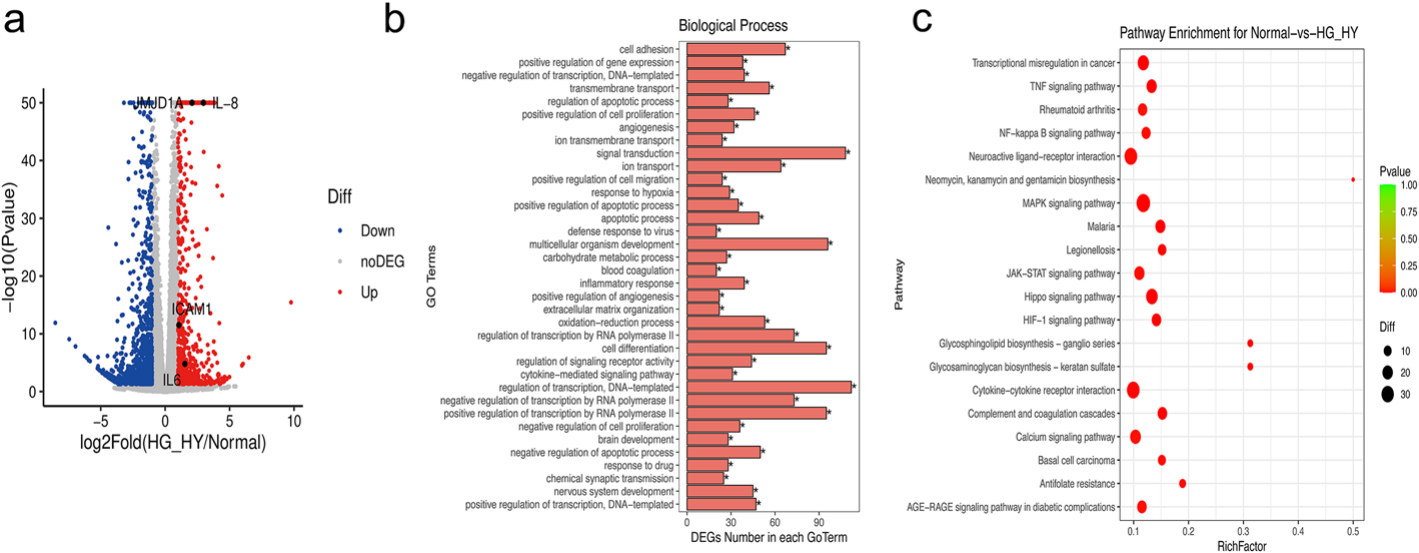
Gene expression of HUVECs induced by high glucose and hypoxia exposure by RNA-sEq. **a** Volcano plot was used to show the difference in gene expression values between the control group and the high glucose hypoxia group. Grey dotted line indicates the threshold for *p* < 0.05 and log FC < 2. Blue and red points represent down-regulated and up-regulated DEGs respectively. GO (**b**) and KEGG enrichment analysis (**c**) of differentially expressed genes (correct p < 0.05). Biological processes are indicated in red, and red bubbles show KEGG pathways

DEGs were analyzed using DEseq2. When the threshold was adjusted to a *p*-value < 0.05, 1353 DEGs were selected and of these 554 genes were up-regulated, while 799 were down-regulated. DEGs are listed in Additional file 3: Table S2. Importantly, IL-8, ICAM-1, and IL-6 were significantly up-regulated, induced by high glucose and hypoxia (Fig. 5a).

**Fig. 6:**
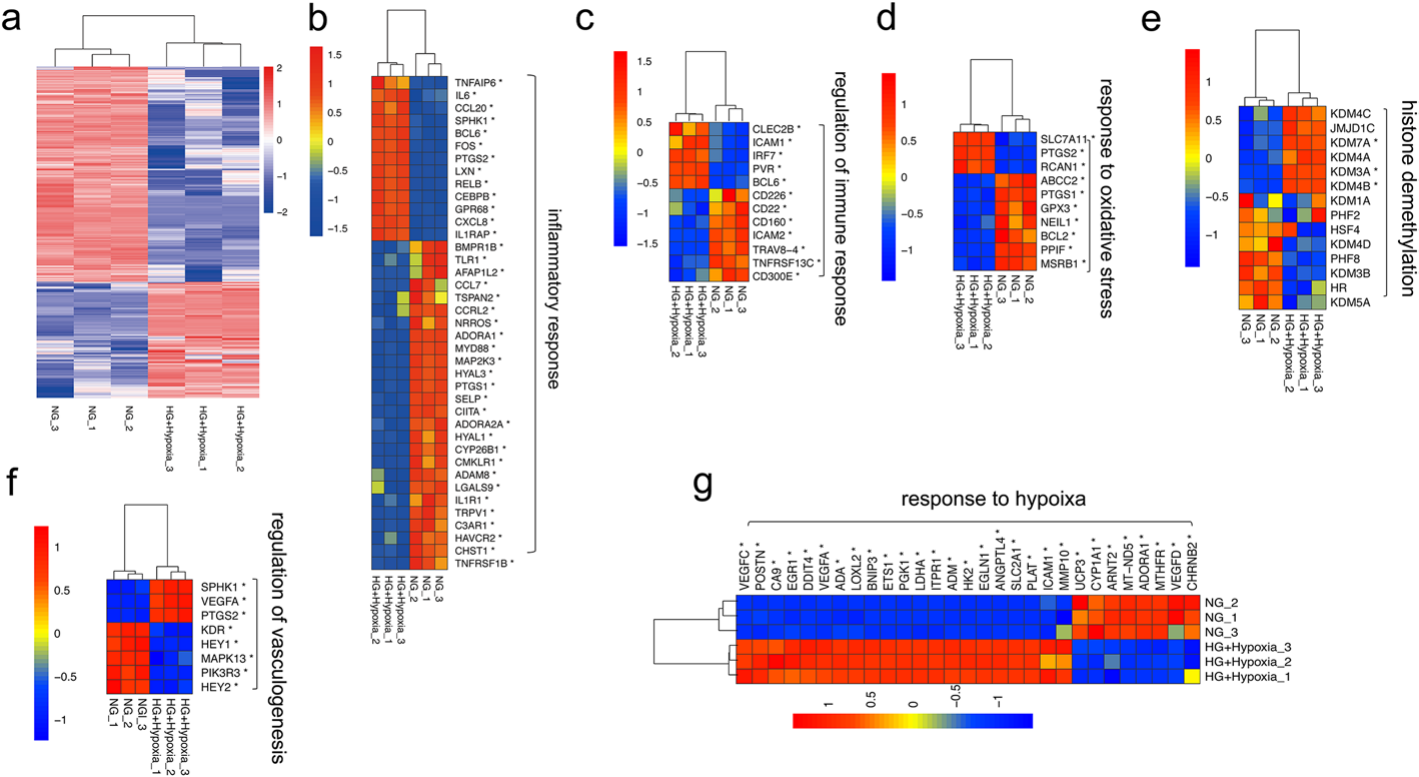
Heatmaps of HUVECs induced by high glucose and hypoxia exposure by RNA-sEq. **a** Heatmaps of differentially expressed genes related to the inflammatory response (**b**), regulation of immune response (**c**), response to oxidative stress (**d**), histone demethylation (**e**), response to angiogenesis (**f**), response to hypoxia (**f**), and regulation of angiogenesis (**g**)

To examine processes and pathways that may be altered upon exposure to high glucose and hypoxia, Gene Ontology (GO) and Kyoto Encyclopedia of Genes and Genomes (KEGG) enrichment analysis was performed. Dozens of biological processes (BPs) were identified, including the inflammatory response, regulation of immune response, response to hypoxia, response to oxidative stress, regulation of angiogenesis, and histone demethylation (Fig. 5b and Additional file 4: Table S3). Regulation of transcription, DNA-template, multicellular organism development, cell differentiation, positive regulation of transcription by RNA polymerase II, negative regulation of transcription by RNA polymerase II, cell adhesion, ion transport, transmembrane transport, angiogenesis, and response to hypoxia were the top 10 enriched BP terms. These results showed that the BPs that involved cell regulation of transcription were unbalanced, especially in BPs including angiogenesis and response to hypoxia, and cell adhesion. The top 30 GO terms with the highest enrichment factor are shown in Fig. 5b. We performed the KEGG pathway enrichment analysis to gain further insights into the function of genes and their interaction for high glucose and hypoxia in HUVECs, and found 322 pathway terms including 55 pathway terms with *p* < 0.05. The top 10 pathways with the greatest enrichment were the MAPK signaling pathway, transcriptional mis-regulation in cancer, human T-cell leukemia virus 1 infection, cell adhesion molecules, cytokine-cytokine receptor interaction, Hippo signaling pathway, HIF-1 signaling pathway, TNF signaling pathway, advanced glycation end products, and the receptor for advanced glycation end products (AGE-RAGE) signaling pathway in diabetic complications and cellular senescence. The top 20 enriched pathways are presented in Fig. 5c. Further, the specific genes associated with signaling pathways or biological processes including the associated inflammatory response, regulation of the immune response, response to hypoxia, response to oxidative stress, regulation of angiogenesis, and histone demethylation in high-glucose and hypoxic conditions are shown in Fig. 6a–g; they were consistent with previous studies.

### Inhibition of HIF-1α decreased inflammation and oxidative stress in HUVECs via JMJD1A induction by exposure to high glucose and hypoxia

**Fig. 7:**
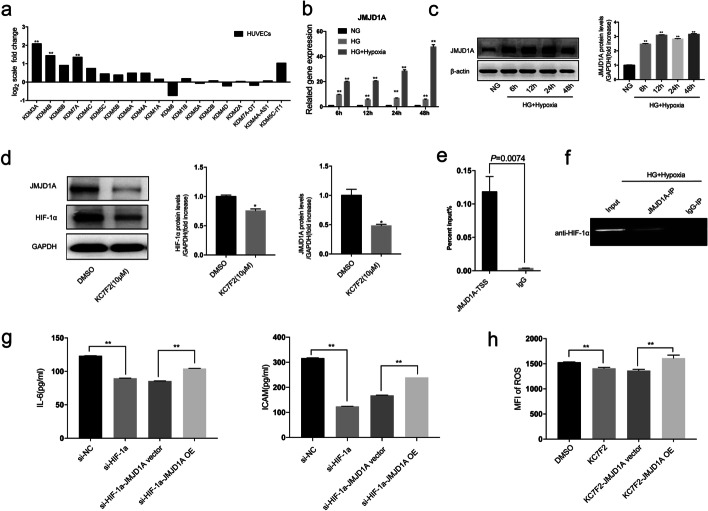
Inhibition of HIF-1α decreases inflammation and oxidative stress in HUVECs via JMJD1A induced by high glucose and hypoxia. **a** Quantitative gene expression changes of JMJD families under high glucose and hypoxia. Cells were treated with high glucose and hypoxia for 6, 12, 24, and 48 h, qRT-PCR (**b**) and western blotting (**c**) showing the expression of JMJD1A. **d** Cells were treated with or without KC7F2 (10 µM) before exposure to high glucose and hypoxia for 48 h. The total proteins of HIF-1α and JMJD1A were collected and analyzed using western blotting as described under Materials and Methods. The relative densities of HIF-1α and JMJD1A were calculated according to GAPDH. **e** Cells were treated with high glucose and hypoxia for 24 h. ChIP assay showing HIF-1α bound to the JMJD1A promoter *in vitro*. The promoter regions of JMJD1A (–2.5 kb to –500 bp) were amplified using the input and immunoprecipitated DNA as templates. **g** Cells were treated with si-HIF-1α/KC7F2 and JMJD1A overexpression induced by high glucose and hypoxia for 48 h. ELISA and flow cytometry showing the secretion of (**g**) IL-6, ICAM-1, and **h** ROS. n = 3; *p < 0.05 and **p < 0.01 vs. DMSO. DMSO-treated cells, DMSO; HIF-1α inhibitors KC7F2 (10 µM)-treated cells, KC7F2 (10 µM); HG, high glucose; HG + Hypoxia, combined stimulus with high glucose and hypoxia; NG, control

We screened the expression of H3K9 demethylating enzymes of the JMJD families by RNA-sEq. Of these, three demethylating enzymes, KDM3A, KDM4B, and KDM7A, were differentially expressed between the two groups. JMJD1A (KDM3A) showed more than a 2-fold increase under high-glucose and hypoxic conditions in HUVEC (Fig. 7a). The differential expression for JMJD1A was further confirmed by qRT-PCR (Fig. 7b) and western blotting (Fig. 7c). These data suggested that JMJD1A was up-regulated in the high-glucose and hypoxic conditions and might be involved in the specific regulation of HUVEC injury. Next, to analyze the pathogenic role of JMJD1A in vascular injuries induced by HIF-1α under high-glucose and hypoxic conditions, inhibition of HIF-1α using KC7F2 and a si-HIF-1α assay was performed. Our study showed that high-glucose and hypoxia stimulated the expression of HIF-1α and JMJD1A, which was attenuated by pre-treatment with KC7F2 (Fig. 7d). Under the same conditions, a CHIP-qPCR assay was also performed in which chromatin was immunoprecipitated with an anti-HIF-1α antibody and the JMJD1A promoter region (from − 432 bp to − 371 bp) was amplified by PCR. As expected, the conventional CHIP-qPCR assay in HUVECs confirmed that JMJD1A promoter fragments containing potential hypoxia responsive element (HRE) sites could be more significantly immunoprecipitated by a specific HIF-1α antibody than by the anti-lgG antibody (Fig. 7e–f). The ameliorative effects of si-HIF-1α and KC7F2 on ROS and inflammation cytokines, specifically IL-6 and ICAM-1 in high-glucose and hypoxia-induced HUVECs, respectively, were reversed by JMJD1A overexpression (Fig. 7g–h). These data suggested that HIF-1α could reduce the inflammatory and oxidative stress levels in a JMJD1A-dependent manner in the presence of high glucose and hypoxia.

### Knockdown of JMJD1A inhibited expression of inflammation and oxidative stress in HUVECs after hyperglycemia and hypoxia stimulus

**Fig. 8:**
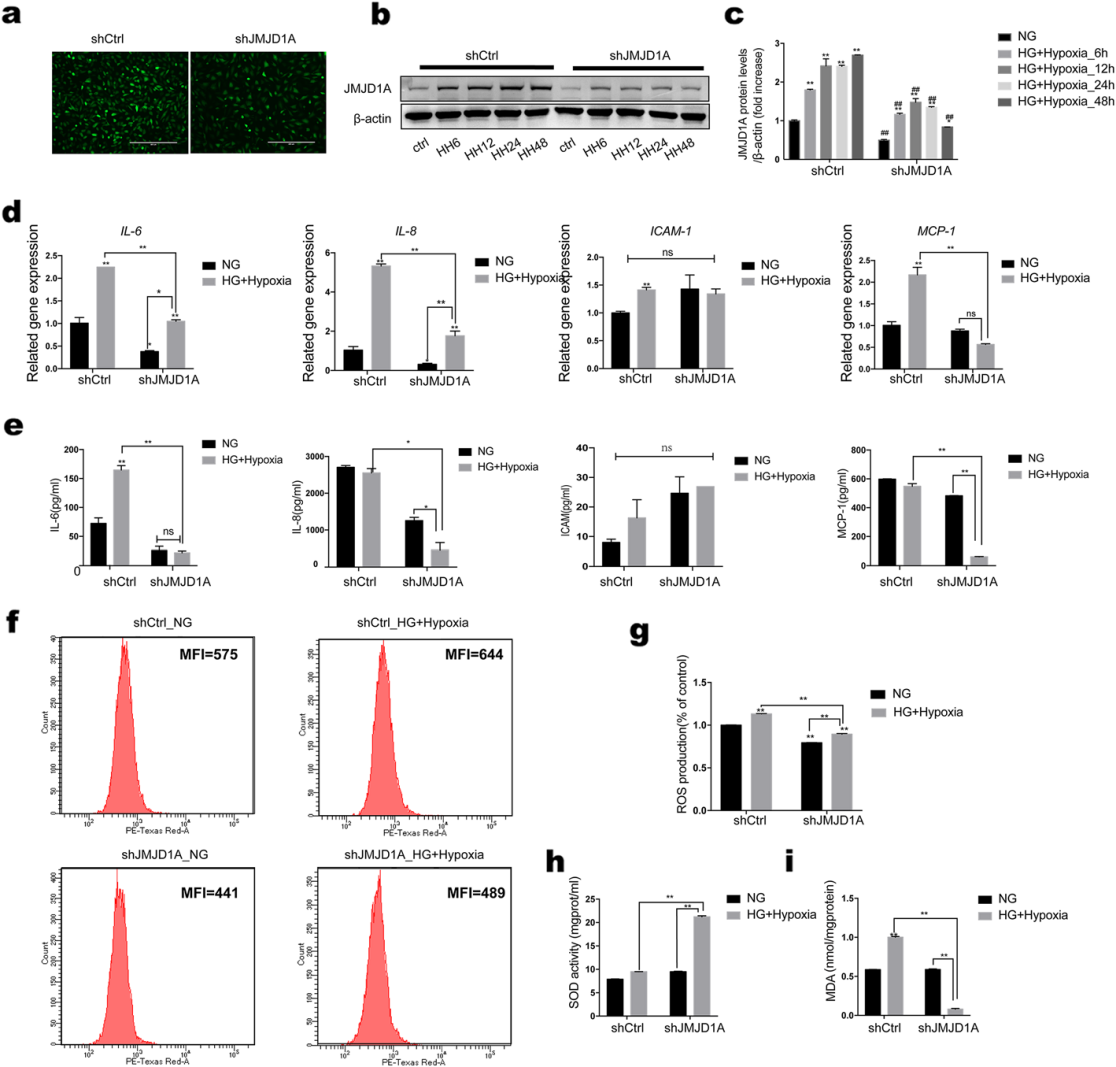
Knockdown of JMJD1A in HUVECs decreases inflammation and oxidative stress injury induced by high glucose and hypoxia. **a** Knockdown of JMJD1A under high-glucose and hypoxic conditions in HUVECs. Western blotting analysis of JMJD1A levels (**b**, **c**) in HUVECs stably transduced with shJMJD1A and control scrambled shRNA (shCtrl), and cultured in normal or high-glucose and hypoxic conditions for 6, 12, 24, and 48 h. Cells were transfected with control shRNA or shJMJD1A for 48 h following combined stimulation, and qPCR (**d**) and ELISA for IL-6, IL-8, ICAM-1, and MCP-1 (**e**) were performed. Downregulation of JMJD1A decreased the secretion of IL-6, IL-8, and MCP-1, but did not affect ICAM-1 secretion. Assays for ROS (**f** and **g**), MDA (**h**), and SOD activity (**i**) are shown compared with controls. JMJD1A shRNA decreased the expression of ROS and MDA level, and increased SOD activity markedly compared with control shRNA-transfected cells. n=3; *p<0.05 and **p<0.01 vs. control shRNA-transfected cells. HG, high glucose; HG+Hypoxia, combined stimulus with high glucose and hypoxia; NG, control; shCtrl, control shRNA

Although it is known how inflammatory and oxidative stress genes function in the high-glucose and hypoxic environment of diabetic vascular disease, it is not clear whether the histone H3K9 demethylation enzyme JMJD1A regulates this process. Therefore, we aimed to analyze the effects of JMJD1A on inflammatory proteins including IL-6, IL-8, ICAM-1, and MCP-1 under hyperglycemia and hypoxic stimulation of HUVECs. We transduced a green fluorescent protein (GFP)-scramble control and a GFP-shJMJD1A into cells, using a lentiviral vector (Fig. 8a). Changes in expression of JMJD1A were also examined under high-glucose and hypoxic conditions for 6, 12, 24, and 48 h to identify the reduction compared with GFP-scrambled cells (Fig. 8b–c). We found that although the downregulation of JMJD1A in cells exposed to the combined stimulus did not affect ICAM-1 secretion, significant decreases were observed in IL-6, IL-8, and MCP-1 secretion (0.46-, 0.33-, and 0.56-fold at the mRNA level and 0.13-, 0.18-, and 0.11-fold at the protein level, respectively), compared with control shRNA transfection (Fig. 8d–e). We therefore inferred that JMJD1A played an important role in co-stimulation-induced injury through the effect on the secretion or function of inflammatory proteins.

Next, we tested whether JMJD1A modulated cell oxidative stress under this condition. The downregulation of JMJD1A reduced ROS and MDA production by 0.75- and 0.58-fold compared with control shRNA-transfected cells, and increased SOD activity by 2.24-fold (Fig. 8f–i). These results suggested that JMJD1A inhibited oxidative stress in the cells exposed to combined stimulus-mediated injury. Overall, these data supported the hypothesis that JMJD1A might accelerate cell inflammation and oxidative stress under the combined high-glucose and hypoxic-induced injury in HUVECs.

### RNA-seq revealed that JMJD1A knockdown may ameliorate the high-glucose and hypoxia-induced endothelial injury via FOS/FOSB

**Fig. 9:**
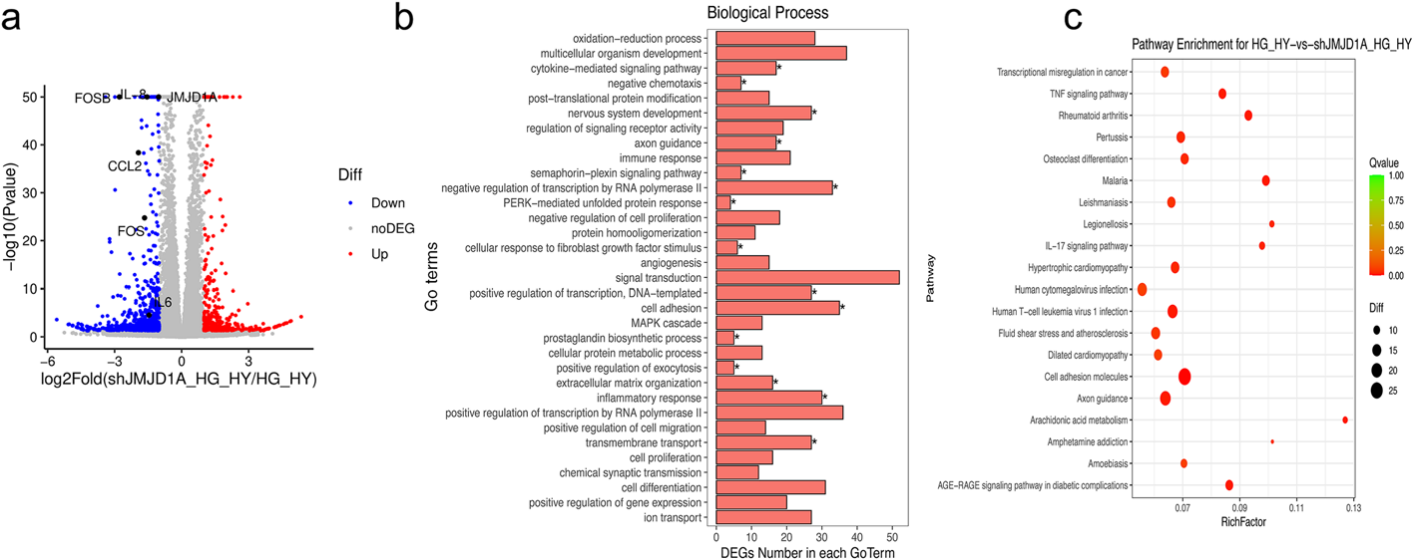
Gene expression of shJMJD1A HUVECs induced by high glucose and hypoxia exposure by RNA-sEq. **a** Volcano plot was used to show the difference in gene expression values between the shCtrl and shJMJD1A induced by high glucose and hypoxia. Grey dotted line indicates the threshold for *p* < 0.05 and log FC < 2. Blue points represent down-regulated and red points represent up-regulated differentially expressed genes. GO (**b**) and KEGG enrichment analysis (**c**) of differentially expressed genes (correct_*P ≤* 0.05). Biological processes are indicated in red, and red bubbles show KEGG pathway

Although JMJD1A expression was very weak in normal oxygen, it markedly increased under high-glucose and hypoxic conditions. This led us to investigate the transcription factors involved in JMJD1A induction under high glucose and hypoxia. In analysis by RNA-seq, using a threshold adjusted to *p* < 0.05, 777 DEGs were selected, of which 302 genes were up-regulated, while 475 were down-regulated in shJMJD1A-treated HUVECs compared with controls transfected with shRNA under high-glucose and hypoxic conditions (Fig. 9a). The DEGs are listed in Additional file 5: Table S4. Further, we performed a comprehensive expression analysis of genes that reversed the high-glucose and hypoxia-induced endothelial injury in the shJMJD1A-transduced HUVECs.

**Fig. 10:**
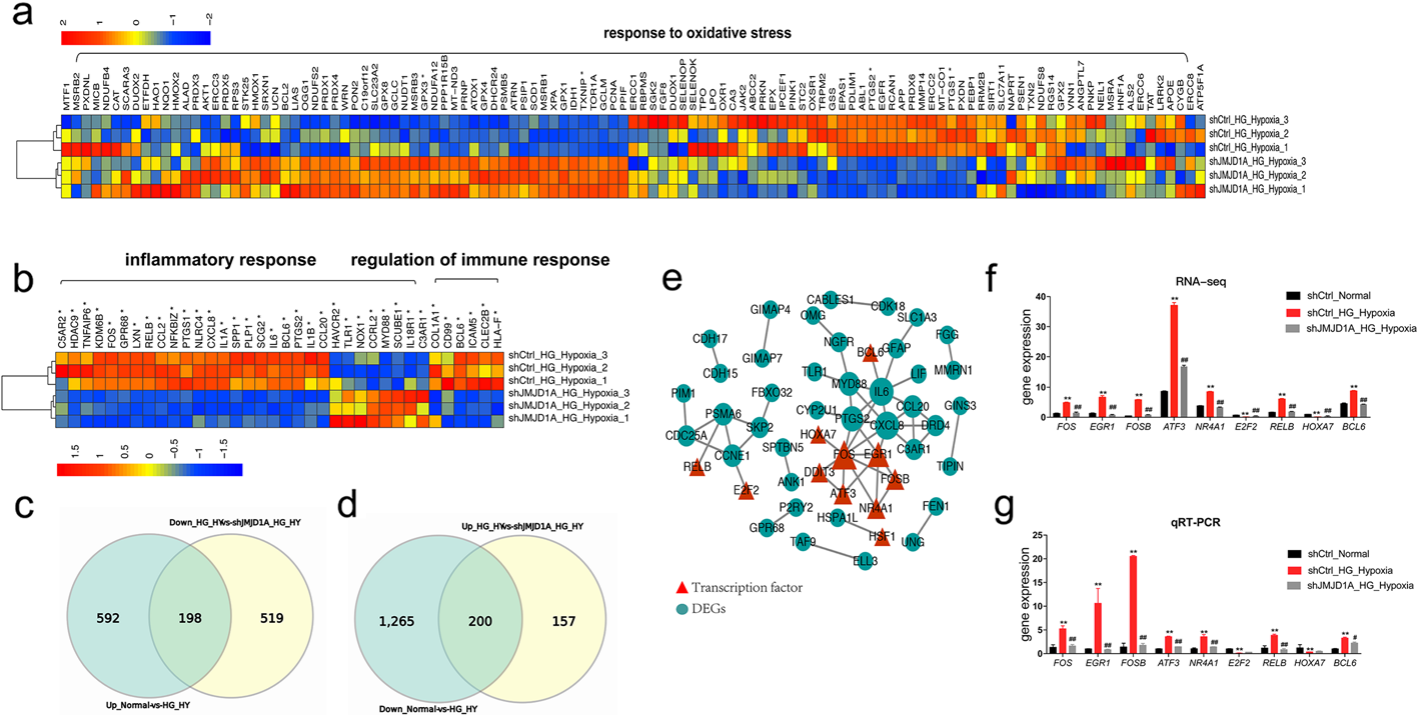
RNA-seq revealed that JMJD1A knockdown ameliorates injury by FOS/FOSB under high glucose and hypoxia. **a**–**b** Heatmaps of differentially expressed genes related to response to oxidative stress (**a**) and inflammatory response, regulation of immune response (**b**). **c**–**d** Venn diagram shows genes regulated by JMJD1A only under high glucose and hypoxia in HUVECs. **e** PPI network of the high-glucose- and hypoxia-induced HUVECs by silencing JMJD1A. The size of the nodes was positively related to the degree of nodes; red triangles indicate transcription factors, while blue nodes indicate DEGs. **f**–**g** Results of RNA-seq (**f**) and qRT-PCR (**g**) for core transcription factors. PPI, protein–protein interaction; HG, high glucose; HG + Hypoxia, combined stimulus with high glucose and hypoxia; NG, control; shCtrl, control shRNA

To further understand the association between JMJD1A and high-glucose and hypoxia-induced EC injury in HUVECs, we screened genes whose expression increased and decreased more than 2-fold in the sample and performed a GO analysis. Our findings showed that the inflammatory response, oxidation-reduction processes, cell adhesion, negative regulation of cell proliferation, angiogenesis, mitogen-activated protein kinase (MAPK) cascade, positive regulation of cell migration, positive regulation of gene expression, axon guidance, and cellular responses to fibroblast growth factor stimulus were the Top 10 enriched BP terms (Additional file 6: Table S5). These results showed that the cell response was unbalanced, especially in the inflammatory response, in oxidation-reduction processes, and in cell adhesion. The top 30 GO terms with the highest enrichment factor are shown in Figs. 9b and 10a–b. We performed KEGG pathway enrichment analysis to provide further insights into the function of genes and their interaction for cells harboring knockdown of JMJD1A, and found 301 pathway terms including 57 pathway terms with a *p* < 0.05. The top 10 pathways showing the greatest enrichment were cell adhesion molecules, phosphatidylinositol 3-kinase (PI3K)-Akt signaling pathway, axon guidance, human cytomegalovirus infection, human T-cell leukemia virus 1 infection, fluid shear stress, atherosclerosis, pertussis, hypertrophic cardiomyopathy, transcriptional mis-regulation in cancer, and focal adhesion pathways. The top 20 enriched pathways were presented in Fig. 9c.

Next, we identified 398 genes that showed increased expression under high-glucose and hypoxic conditions, which were downregulated by shJMJD1A, or genes with decreased expression in high glucose and hypoxia that were upregulated by shJMJD1A (Fig. 10c–d and Additional file 7: Table S6). Next, the 398 significant JMJD1A-related genes were used to construct a protein–protein interaction (PPI) network. As shown in Fig. 10e, the PPI network consisted of 50 nodes and 56 interactions. Many genes were highly connected, including: *FOS*, *EGR1*, *FOSB*, *ATF3*, *NR4A1*, *E2F2*, *RELB*, *HOXA7*, and *BCL6*. The relative gene expression levels of the core transcription factors were analyzed by qRT-PCR and RNA-seq under a hypoxic and high-glucose environment. The results indicated that the expression of *FOS*, *EGR1*, *FOSB*, *ATF3*, *NR4A1*, *RELB*, and *BCL6* in the hypoxia and high-glucose treated group increased significantly in comparison to that of the control group, and decreased in the shJMJD1A group (Fig. 10f–g). Details for the top 10 JMJD1A-related genes and transcription factors are listed in Additional file 8: Table S7.

## Discussion

As far as we know, our study is the first to present the following findings: (i) HIF-1α/JMJD1A expression was induced by exposure to high-glucose and hypoxic conditions in a time-dependent manner and was positively associated with inflammatory and oxidative stress injury in HUVECs; and (ii) downregulation of HIF-1α/JMJD1A inhibited the excessive production of inflammatory and oxidative stress of HUVECs induced by high glucose and hypoxia.

Endothelial dysfunction induced by hyperglycemia plays a critical role in the occurrence and development of diabetic vascular complications [46, 47]. However, the exact mechanism of endothelial dysfunction caused by hyperglycemia has not been fully studied. In vitro, the expression of HIF-1α and JMJD1A in ECs was upregulated by high glucose and hypoxia in the present study, indicating that the two may serve as important factors of endothelial dysfunction in diabetes. The precise mechanism by which high glucose and hypoxia regulate HIF-1α and JMJD1A expression in ECs requires further study.

Multiple lines of evidence have indicated that high glucose and hypoxia cause chronic inflammation and oxidative stress in vivo and in vitro, processes which are considered to be closely associated with HIF-1α expression [48]. HIF-1α binds to HREs on the promoter region of many genes that regulate the cellular response not only under hypoxic conditions but also inflammatory conditions [49, 50]. Herein, we present evidence that the combined exposure of HUVECs to high glucose and hypoxia induced HIF-1α expression and increased the cell oxidative stress and inflammatory progression. Interestingly, the downregulation of HIF-1α reduced the cell oxidative stress and inflammatory progression after exposure to high-glucose and hypoxic conditions in HUVECs. In addition, we observed excessive production of inflammatory bio-mediators and oxidative stress indicators such as ROS and MDA, which indicated a pro-oxidant shift in the redox balance of cellular lipids [51, 52]. Upon redox stress, an important defense is provided by the SOD enzymes, which ensure a rapid flux in the removal process of the redox couple O_2_^−^/H_2_O_2_ [53, 54]. This suggests that oxidative stress and inflammation are implicated in the pathophysiology of diabetes. Notably, HIF-1α can facilitate oxidative stress and inflammatory progression. The underlying mechanisms behind these effects in HIF-1α activity in facilitating cell oxidative stress and inflammatory progression remain unclear.

To this aim, we analyzed mRNA expression profiles by high-throughput RNA sequencing in HUVECs from the control and high-glucose and hypoxic conditions. A total of 1353 DEGs were identified, including 554 up-DEGs and 799 down-DEGs. Bioinformatics analysis provided us with a better understanding of the mechanism of high-glucose- and hypoxia-induced injury in HUVECs. In our study, we identified hundreds of genes and pathways that might play a role in the high-glucose and hypoxia setting in HUVECs, notably genes related to the inflammatory response, response to hypoxia, and response to oxidative stress. By pathway enrichment analysis, 14 DEGs were shown to be linked to histone demethylation: *JMJD1A*, *KDM4B*, *JMJD1C*, *KDM7A*, *KDM4C*, *KDM4A*, *KDM1A*, *HR*, *PHF8*, *PHF2*, *HSF4*, *KDM5A*, *KDM4D*, and *KDM3B*. We also identified 21 genes of the JMJD family, i.e. *KDM1A*, *KDM7A*, *KDM4A*, *KDM5A*, *KDM2B*, *KDM4C*, *KDM3A*, *KDM5B*, *KDM3B*, *KDM5C*, *KDM4B*, *KDM6B*, *KDM6A*, *KDM8*, *KDM1B*, *KDM2A*, *KDM4D*, *KDM5C-IT1*, *KDM4E*, *KDM4A-AS1*, and *KDM7A-DT*, and found that the expression of *JMJD1A* was much higher than the expression of other members of the JMJD gene family.

HIF-1α is an initiating factor that regulates myocardial hypoxia and triggers the endogenous inflammatory mechanism. Several transcriptional targets of HIF-1α have been identified; however, in the progression of oxidative stress and inflammation, most of the “high-glucose and hypoxia-specific” transcriptional targets of HIF-1α are unknown. Our study verified that upregulation of *JMJD1A* in HUVECs exposed to hypoxia and high-glucose conditions in vitro was abrogated when HIF-1 signaling was blocked by the inhibitor KC7F2. Further, our data confirmed that JMJD1A was a target of HIF-1α in oxidative stress and inflammation under high glucose and hypoxia by the CHIP-PCR assay, while the biological effects of HIF-1α on oxidative stress and inflammatory cytokines in high-glucose- and hypoxia-induced HUVECs were reversed by JMJD1A overexpression. Further, *JMJD1A* knockdown may decrease inflammatory injury by downregulating the secretion of IL-6, IL-8, and MCP-1, reducing oxidative stress progression by suppressing the levels of ROS and MDA, and by increasing the SOD activity. These data indicated that knockdown of HIF-1α/JMJD1A could protect the macrovascular ECs against both inflammatory and oxidative stress using HUVECs as an in vitro model. Several reports have described the role of JMJD1A in angiogenesis and cell growth under hypoxia. For example, Adam et al. reported that JMJD1A acted as a signal amplifier to promote hypoxia gene expression in hypoxia, and ultimately enhanced tumor growth in both renal and colon carcinoma cell lines. It has also been reported that in solid tumors, such as breast cancer and rectal cancer, high JMJD1A expression is associated with a poor prognosis. As described earlier, JMJD1A serves as an oncogenic factor in tumors subjected to hypoxic conditions. Although JMJD1A is known to be induced by hypoxia in a variety of cancers, no association has yet been identified between inflammation/oxidative and JMJD1A in high glucose and hypoxia in ECs. Nevertheless, our present study showed that JMJD1A knockdown rescued these effects almost completely. Our study demonstrated the ability of JMJD1A to decrease inflammatory and oxidative stress under the conditions of high glucose and hypoxia in HUVECs. The underlying mechanisms behind these effects of JMJD1A in rescuing inflammation and oxidative stress remain unclear.

In the current study, the PPI network showed that FOS/FOSB might be the most important transcriptional factor associated with JMJD1A expression in the combined stimulation of high glucose and hypoxia. RNA sequencing demonstrated that there was increased expression of FOS and FOSB in the HUVECs exposed to the combined stimulation of high glucose and hypoxia. The permanent downregulation of JMJD1A decreased the levels of FOS and FOSB simultaneously. Previous studies have shown that FOS/FOSB activates the expression of IL-6, IL-8, ICAM-1, and MCP-1 in response to hypoxia [55, 56]. Based on these findings, we hypothesized that JMJD1A regulated injury induced by high glucose and hypoxia by influencing the functions of FOS/FOSB. In the future, we will try to clarify the role of FOS or FOSB in the cyto-protection of HUVEC induced by high glucose and hypoxia.

Altogether, this study suggested for the first time that decreasing the expression of HIF-1α could suppress oxidative stress and inflammatory progression via JMJD1A in HUVECs induced by hypoxia and high glucose. This subsequently affected cellular oxidative stress and inflammation, and ultimately led to the development of diabetic vascular complications. The HIF-1α/JMJD1A pathway might act as a novel regulator of oxidative stress and inflammatory-related events in response to diabetic vascular injury, thus contributing to the pathological progression of diabetes and vascular disease.

## Supplementary Information

**Additional file 1: Fig. S1.** Stimulation with high glucose decreases cell survival in HUVECs. Cells treated with various concentrations of glucose for (a) 6, (b) 12, (c) 24, or (d) 48 h reduced cell viability. n = 3; *p < 0.05 and **p < 0.01 vs. control; NG, control.

**Additional file 2: Table S1.** Primers used for quantitative RT-PCR.

**Additional file 3: Table S2.** List of genes that are differentially expressed after 48-h exposure to high-glucose and hypoxia treatment (p < 0.05).

**Additional file 4: Table S3.** GO enrichment analysis of differentially expressed genes exposed to high-glucose and hypoxic conditions in HUVECs.

**Additional file 5: Table S4.** List of genes that are differentially expressed after JMJD1A knockdown of HUVECs exposed to high-glucose and hypoxia treatment (p < 0.05).

**Additional file 6: Table S5.** GO enrichment analysis of differentially expressed genes in shJMJD1A HUVECs in high-glucose and hypoxic conditions.

**Additional file 7: Table S6.** List of JMJD1A-related genes that are differentially expressed on exposure to high-glucose and hypoxic treatment (p < 0.05).

**Additional file 8: Table S7.** List of transcription factors that are differentially expressed after JMJD1A knockdown in HUVECs exposed to high-glucose and hypoxia treatment (P < 0.05).

## Data Availability

The data and materials in the current study are available from the corresponding author on reasonable request.

## Abbreviations

EC: Endothelial cell
HIF-1α: Hypoxia-inducible factor-1 alpha
NF-κB: Nuclear factor kappa-B
HUVECs: Human umbilical vein endothelial cells
RNA-seq: RNA sequencing
si-HIF-1α: Small interfering HIF-1α
JMJD1A: Jumonji domain-containing protein 1A
qRT-PCR: Quantitative reverse transcription-polymerase chain reaction
ELISA: Enzyme-linked immunosorbent assay
Ch-IP: Chromatin immunoprecipitation
DM: Diabetes
CVD: Cardiovascular disease
IL: Interleukin
IDF: International Diabetes Federation
MCP-1: Monocyte chemoattractant protein 1
ICAM-1: Intercellular adhesion molecule-1
ROS: Reactive oxygen species
MDA: Malondialdehyde
SOD: Superoxide dismutase
H3K9 me1/2: H3 lysine 9 dimethyl and monomethyl
HRE: Hypoxia responsive element
FBS: Fetal bovine serum
DMEM/F12: Dulbecco’s modified Eagle’s medium F12
CCK-8: Cell Counting Kit 8
MFI: Mean fluorescence intensity
BCA: Bicinchoninic acid
BH: Benjamini–Hochberg
DEGs: Differentially expressed genes
FDR: False discovery rate
GO: Gene Ontology
KEGG: Kyoto Encyclopedia of Genes and Genomes
BP: Biological processes
AGE-RAGE: Advanced glycation end products and receptor for advanced glycation end products
GFP: Green fluorescent protein
MAPK: Mitogen-activated protein kinases
PI3K: Phosphatidylinositol 3-kinase
PPI: Protein–protein interaction
