# Supplementary figures and images for: HIF-1α/JMJD1A signaling regulates inflammation and oxidative stress following hyperglycemia and hypoxia-induced vascular cell injury
Source: Cell Mol Biol Lett. 2021 Sep 3;26:40. doi: 10.1186/s11658-021-00283-8 (PMC8414688; doi:10.1186/s11658-021-00283-8)

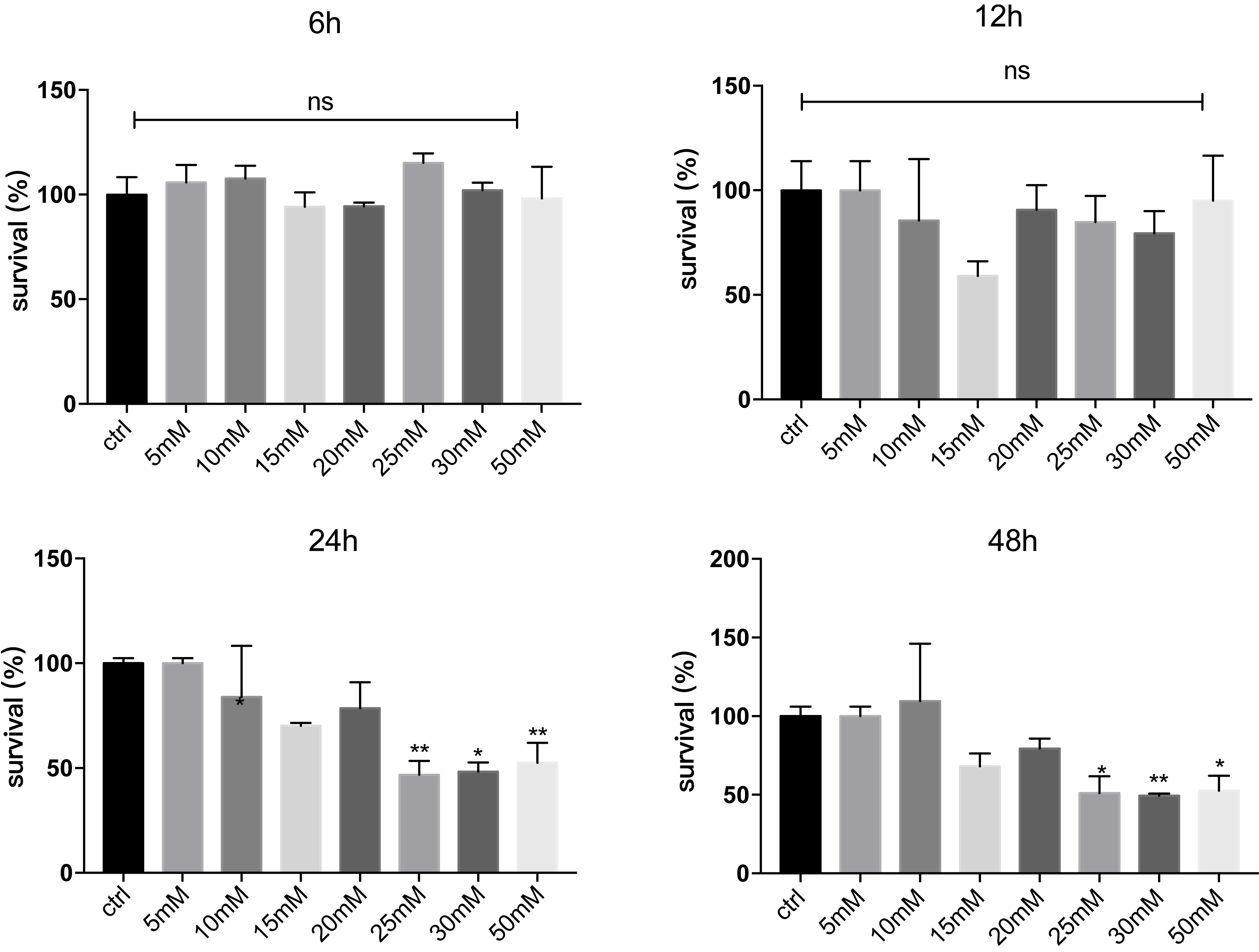

Supplement: Supplementary file 1 — Additional file 1: Fig. S1. Stimulation with high glucose decreases cell survival in HUVECs. Cells treated with various concentrations of glucose for (a) 6, (b) 12, (c) 24, or (d) 48 h reduced cell viability. n = 3; *p < 0.05 and **p < 0.01 vs. control; NG, control. [file 11658_2021_283_MOESM1_ESM.tif]
