# Supplementary material for: HIF-1α/JMJD1A signaling regulates inflammation and oxidative stress following hyperglycemia and hypoxia-induced vascular cell injury
Source: Cell Mol Biol Lett. 2021 Sep 3;26:40. doi: 10.1186/s11658-021-00283-8 (PMC8414688; doi:10.1186/s11658-021-00283-8)
Supplement: Supplementary file 2 — Additional file 2: Table S1. Primers used for quantitative RT-PCR. [file 11658_2021_283_MOESM2_ESM.docx]

| Name | Sequence (5′ - 3′) |
| --- | --- |
| β-Actin | Forward: AGGTCGGTGTGAACGGATTTG  Reverse: TGTAGACCATGTAGTTGAGGTCA |
| IL-6 | Forward: CCGAAGCAAACATCACATTCA  Reverse: GGTCTAAAGGCTCCGGGCT |
| ICAM-1 | Forward: GTAGCCCACGTCGTAGCAAA  Reverse: ACAAGGTACAACCCATCGGC |
| MCP-1 | Forward: GAGGACATGAGCACCTTCTTT  Reverse: GCCTGTAGTGCAGTTGTCTAA |
| IL-8 | Forward: CCCTTTGCTATGGTGTCCTTTC  Reverse: AGGATCTCCCTGGTTTCTCTTC |
| HIF-1α | Forward: GAACGTCGAAAAGAAAAGTCTCG  Reverse: CCTTATCAAGATGCGAACTCACA |
| JMJD1A | Forward: GTGTGTGGAATTTGATGGG  Reverse: CAGCTTTGTCCAACAGAGG |
| FOS | Forward: GGGGCAAGGTGGAACAGTTAT  Reverse: CCGCTTGGAGTGTATCAGTCA |
| EGR1 | Forward: ACCCCTCTGTCTACTATTAAGGC  Reverse: TGGGACTGGTAGCTGGTATTG |
| FOSB | Forward: GCTGCAAGATCCCCTACGAAG  Reverse: ACGAAGAAGTGTACGAAGGGTT |
| ATF3 | Forward: CCTCTGCGCTGGAATCAGTC  Reverse: TTCTTTCTCGTCGCCTCTTTTT |
| NR4A1 | Forward: ATGCCCTGTATCCAAGCCC  Reverse: GTGTAGCCGTCCATGAAGGT |
| E2F2 | Forward: CGTCCCTGAGTTCCCAACC  Reverse: GCGAAGTGTCATACCGAGTCTT |
| BCL-6 | Forward: ACACATCTCGGCTCAATTTGC  Reverse: AGTGTCCACAACATGCTCCAT |
| HOXA7 | Forward: TCGTATTATGTGAACGCGCTT  Reverse: CAAGAAGTCGGCTCGGCATT |
| RELB | Forward: CCATTGAGCGGAAGATTCAACT  Reverse: CTGCTGGTCCCGATATGAGG |

**Additional file 2: Supplementary Table S1.** Primers used for quantitative RT-PCR.
